# Epidemiology and Clinical Burden of Malaria in the War-Torn Area, Orakzai Agency in Pakistan

**DOI:** 10.1371/journal.pntd.0004399

**Published:** 2016-01-25

**Authors:** Asad Mustafa Karim, Irfan Hussain, Sumera Kausar Malik, Jung Hun Lee, Ill Hwan Cho, Young Bae Kim, Sang Hee Lee

**Affiliations:** 1 National Leading Research Laboratory, Department of Biological Sciences, Myongji University, Yongin, Gyeonggido, Republic of Korea; 2 Department of Zoology, Kohat University of Science and Technology, Kohat, Pakistan; 3 Department of Biotechnology, Quaid-i-Azam University Islamabad, Islamabad, Pakistan; 4 University of British Columbia, Vancouver, Canada; 5 Department of Natural Science, North Shore Community College, Danvers, Massachusetts, United States of America; University of California San Diego School of Medicine, UNITED STATES

## Abstract

**Background:**

Military conflict has been a major challenge in the detection and control of emerging infectious diseases such as malaria. It poses issues associated with enhancing emergence and transmission of infectious diseases by destroying infrastructure and collapsing healthcare systems. The Orakzai agency in Pakistan has witnessed a series of intense violence and destruction. Military conflicts and instability in Afghanistan have resulted in the migration of refugees into the area and possible introduction of many infectious disease epidemics. Due to the ongoing violence and Talibanization, it has been a challenge to conduct an epidemiological study.

**Methodology/Principal Findings:**

All patients were sampled within the transmission season. After a detailed clinical investigation of patients, data were recorded. Baseline venous blood samples were taken for microscopy and nested polymerase chain reaction (nPCR) analysis. *Plasmodium* species were detected using nested PCR (nPCR) and amplification of the small subunit ribosomal ribonucleic acid (ssrRNA) genes using the primer pairs. We report a clinical assessment of the epidemic situation of malaria caused by *Plasmodium vivax* (86.5%) and *Plasmodium falciparum* (11.79%) infections with analysis of complications in patients such as decompensated shock (41%), anemia (8.98%), hypoglycaemia (7.3%), multiple convulsions (6.7%), hyperpyrexia (6.17%), jaundice (5%), and hyperparasitaemia (4.49%).

**Conclusions/Significance:**

This overlooked distribution of *P*. *vivax* should be considered by malaria control strategy makers in the world and by the Government of Pakistan. In our study, children were the most susceptible population to malaria infection while they were the least expected to use satisfactory prevention strategies in such a war-torn deprived region. Local health authorities should initiate malaria awareness programs in schools and malaria-related education should be further promoted at the local level reaching out to both children and parents.

## Author Summary

The malaria epidemic and endemic in Pakistan is a present and ongoing threat to public health which could have an impact in the nearby regions as well. For the first time, we report a clinical assessment of malaria endemicity in the Orakzai Agency, which is Pakistan’s most neglected area due to Talibanization and war in Afghanistan. Febrile patient blood samples of the area were investigated to report the clinical assessment of malaria caused by *Plasmodium vivax* and *P*. *falciparum* infections. The nested polymerase chain reaction (nPCR) examination detected 154 (86%) and 21 (12%) *P*. *vivax* and *P*. *falciparum* infections, respectively. We found worsening hygiene conditions in FATA, likely caused by poor socioeconomics and the collapse of the public health infrastructure. Decompensated shock was a common and prominent clinical feature of malaria among all the clinical presentations caused by both *P*. *vivax* (53%) and *P*. *falciparum* (42.9%). Our results have significant implications for both public health and malaria control in FATA and Pakistan. Our findings illustrate higher prevalence of malaria in children compared to other age groups. Further research on sensible estimates of refugees is required, as well as resistance to anti-malarials.

## Introduction

Disease emergence is influenced by both natural and human factors. Among human activities, military conflicts characterized by war and regional tribal and/or sectarian strife have had huge impact by destroying infrastructure and collapsing healthcare systems. The affected region and the people therein face diverse short-term and long-term consequences. Large population displacements present higher risks of infectious diseases, lack of resources, which is often the case within crowded refugee camps with sanitation issues, increased exposure of the population to disease vectors, and destruction of healthcare systems lead to negative consequences [1]. Moreover, if the situation were to prolong, discontinued public and healthcare professional education investment and lack of proper surveillance and control of the disease should produce more severe and chronic outcomes. These outcomes make the people in the affected and nearby regions become newly vulnerable to a variety of communicable diseases and directly associated with the prevalence and emergence of the infectious diseases [1, 2].

The prolonged Soviet war in Afghanistan between 1979 and 1995 annihilated the malaria vector control programs in the nation, which had been implemented in the 1960s and 1970s. The programs were so successful that Afghanistan had virtually eradicated the disease in the late 1970s [3]. The collapse of the program resulted in the re-emergence of malaria, and turned the region into a malaria endemic area. After the Soviet war, Afghanistan and the nearby Pakistani regions, specifically the Federally Administered Tribal Areas (FATA, a semi-autonomous territory running along the Pakistan–Afghanistan border) transformed into religious fundamentalism where tribe heads lost their authority and the rule of militant groups such as the Taliban started. Moreover, the subsequent 9/11 triggered occupation of the United States initiated a sizable population displacement of the refugees to FATA Pakistan. The border-crossing migration of Afghan refugees has overwhelmed the local public health system, and has caused an malaria epidemic [4]. Many epidemiology studies attributed the roughly 24%–36% increase in malaria prevalence in this region to the post-Soviet war influx of Afghan refugees into FATA [5].

Different researchers reported that permanent residents have low susceptibility and high immunity against malaria as compared to Afghan refugees [6]. The proportion of malarial cases due to *P*. *vivax* is increasing every year. According to the World Health Organization (WHO) report, approximately 75% (previously 64%) of the infections are transmitted through *P*. *vivax*, whereas 25% (previously 36%) are caused by *P*. *falciparum*, which are the two most prevailing species in Pakistan [7]. Currently, the *P*. *vivax* malaria is accounted in 70% of the malaria burden in Pakistan, and FATA being the most impoverished and extremely underdeveloped area in the Pakistan has the highest malaria burden due to the large Afghan refugees and IDPs [8]. Social development gages are appallingly low. There are only 41 hospitals for a population of 3.1 million.

**Fig 1:**
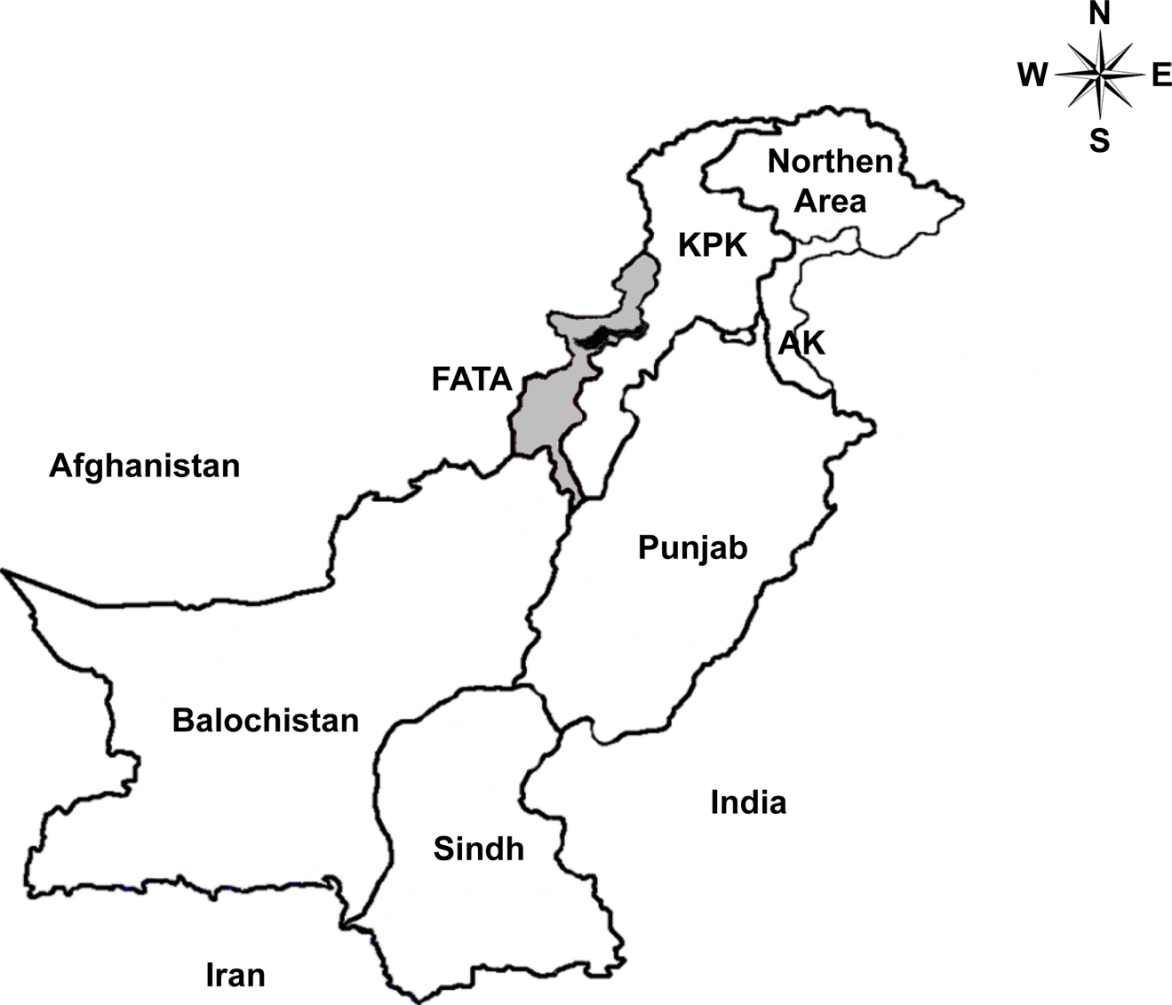
Geographical location of sample collection site (Orakzai Agency: black area) in FATA, Pakistan. FATA (gray): Federally Administered Tribal Areas, KPK: Khyber Pakhtunkhwa, AK: Azad Kashmir.

The Orakzai Agency (Fig 1) has been one of the most neglected areas in FATA during the multi-decade-long military conflicts in the region. The Orakzai tribes have been linked to and have provided safe haven to the Taliban militant groups since late 2001. A large part of the Orakzai Agency soon fell under control of the Taliban. The Agency was once home to Hakimullah Mehsud, the Tehrik-i-Taliban Pakistan chief who led militant operations, targeting hundreds of NATO supply vehicles in 2008 and 2009. The regional medical centers and educational institutions were destroyed by repeated militant attacks [9]. This caused disruption to malaria management efforts as well as the increase of the malaria parasite reservoir in the region. For proper detection and control of malaria, obtaining and analyzing reliable epidemiology data is essential. To the best of our knowledge, however, there has been no report on malaria epidemics and clinical manifestations in Orakzai Agency. It has been a challenge to carry out such studies due to the severity of military conflicts.

We report here the first study of epidemiology and clinical burden of malaria in this war-torn and Talibanized region, Orakzai Agency in Pakistan (the sample collecting region is shown in Fig 1). Keeping in view all the state of affairs and distribution of malaria epidemics in nearby FATA regions, the current study aimed to investigate the epidemiology and clinical manifestations of *P*. *vivax* and *P*. *falciparum* malaria among male and female patients of different age groups. We also report the analytical, epidemiological and clinical differences between *P*. *vivax* and *P*. *falciparum* infections.

## Methods

### Study area and health infrastructure

Orakzai Agency (FATA in Pakistan, Fig 1) is divided into the upper Orakzai and the lower Orakzai. Orakzai is a long neglected area with lack of basic health necessities, occasional engagement of armed insurgents, poor living conditions, limited access to vaccines, limited use of vector control measures, and unequal distribution of economic resources; most importantly, this is the case of approximately 60% of the FATA residents [10]. Furthermore, the medical centers and educational institutions have also been ruined by militant attacks. Healthcare-related non-government organization (NGO) activities are not permitted in FATA. In 2012, according to UNHCR, nearly 758,000 internally displaced persons (IDPs) fled from their homes as a result of security operations in the FATA [11]. More recently, in June 2014, Pakistani military initiated operation against militant groups in FATA resulted in approximately 450,000 IDPs displacement in the Bannu district [12]. Presently, FATA has the highest burden of different infectious diseases, due to the large Afghan refugees and IDPs. The population suffering from or at risk of contracting malaria significantly increased in the FATA as did the malaria parasite reservoir.

### Sample collection

This retrospective case-control study was conducted at the District Headquarters Hospital (DHH) Kalaya in Orakzai Agency, Pakistan between April 2011 and December 2013. Patients presented with major clinical symptoms (fever, headache, cough, dyspnea, vomiting, diarrhea, abdominal pain, and convulsions) of malaria at different partially functional health care centers were referred to the DHH Kalaya. The major clinical symptoms of malaria were based on WHO criteria [8, 13]. All children and adults presenting to hospital were screened for study eligibility and were hospitalized. A total of 216 microscopy-confirmed patients aged 1–60 years were evaluated in the study. Demographic and clinical records were collected upon enrollment, and baseline venous blood samples were collected for further biochemical and molecular analyses. Control group was collected at the same site as uncomplicated cases and used for statistical analyses. To exclude the confounding effect of sex, age and locality, control population was matched by sex, age and locality. Pregnant women were not included in the study. The inclusion criteria for patients were as follows: (i) prostration (unable to sit), (ii) multiple seizures, (iii) impaired consciousness, (iv) multiple convulsions, (v) hyperpyrexia, (vi) anemia, (vii) decompensated shock, (viii) dark urine, (ix) jaundice, (x) hypoglycaemia, (xi) hyperparasitaemia, and (xii) respiratory problems [14]. In severe malaria, the level of impaired consciousness was assessed by computing the Glasgow Coma Scale (GCS) score (<11) in adults or Blantyre Coma Scale Score (<3) in children [14]. For further investigation, studied patients were divided into groups on the basis of their age and sex.

### Clinical assessment

After a detailed clinical investigation of patients, a standardized case report template was designed to compile the complete clinical data of each patient. For children younger than five years, their parents or relatives were asked of their medical history.

### Microbiological diagnosis

Baseline venous blood samples were taken for microscopy and nested polymerase chain reaction (nPCR) analysis. The initial diagnosis of *Plasmodium* spp. infection was made by thick or thin smears. Two slides were made from each patient’s blood and both thick and thin films were prepared on the slides in the DHH Kalaya laboratory. Giemsa-stained thick blood smears of patients were examined using Giemsa stain and the parasitemia quantified independently by two skilled microscopists [15]. A thick smear was considered negative if no parasite was seen in at least 200 fields.

### Isolation of parasite DNA and molecular examination

For molecular analysis, the parasite DNA was extracted from filter papers using the Qiagen DNA extraction kit (QIAGEN, Valencia, CA, USA), following to the manufacturer’s protocol. The *Plasmodium* species were detected using nested PCR (nPCR) and amplification of the small subunit ribosomal ribonucleic acid (ssrRNA) genes using the primer pair set A (5’-TTAAAATTGTTGCAGTTAAAACG-3’ and 3’-CCTGTTGTTGCCTTAAACTTC-5’) for the detection of *P*. *vivax*; primer pair set B (5’-CGCTTCTAGCTTAATCCACAT AACTGATAC-3’ and 3’-ACTTCCAAGCCGAAGCAAAGAAAGTCCTTA-5’)for the detection of *P*. *falciparum*; primer pair set C (5’-CTGTTCTTTGCATTCCTTATGC-3’ and 3’-GTATCTGATCGTCTTCACTCCC-5’) for the detection of *P*. *ovale*; and primer pair set D (5’-GTTAAGGGAGTGAAGACGA-3’ and 3’- TCAGAAACCCAAAGACTTTGATTTCTCAT-5’) for the detection of *P*. *malariae*. PCR reactions were carried out on a thermal cycler (Nyx Technik USA), beginning with 5 minutes at 94°C, followed by 25 cycles of 45 seconds at 94°C, 45 seconds at 58°C, and 5 minutes at 72°C for the first round; 30 cycles of 45 seconds at 94°C, 45 seconds at 65°C, and 2 minutes at 72°C was then performed for the second round. The final cycle was followed by an extension time of 5 minutes at 72°C. The amplified PCR products were analyzed by 2%–2.5% agarose gel electrophoresis stained with ethidium bromide and visualized on the Bio-Rad gel doc system (Bio-Rad Laboratories, Hercules, CA, USA). Due to the absence of well-equipped laboratory facilities in the DHH Kalaya, PCR and biochemical analyses were carried out at the Kohat University of Science and Technology and Kohat hospital.

### Statistical analysis

Statistical analysis was carried out on SPSS version 19. Means, odds ratios with 95% CIs, and χ2 test of independence were calculated when applicable. Statistica (version 12) was used for box-and-whisker plots. In all the studied parameters, a p value of ≤0.05 was considered statistically significant. Patients with prior comorbid conditions were excluded from relevant subanalyses, for example, diabetes mellitus patients were excluded from hypoglycemia analysis. All analyses were repeated after excluding all patients with associated infections and comorbid illnesses.

### Ethics statement

Ethical approval for project activities was provided by the Kohat University of Science and Technology. Written informed consent was obtained from the patients and their parents/guardians before recruitment.

## Results

### General characteristics of study

We collected 216 blood samples from febrile patients between April 2011 and December 2013 and screened for malaria. These febrile samples were collected from the DHH Kalaya in Orakzai Agency, Pakistan, where ordinary disease detection and control activities had been halted due to decade-long military conflicts. Many of the febrile patients were known to be displaced Afghan refugees, but such data was not recorded. A total of 216 blood samples were diagnosed positive by the microscopic examination. As baseline patient demographics are shown in Table 1, 178 of 216 patients were identified by nPCR to have contracted malaria (mean ± SD age 19.9 ± 11.9 years). Among these, we have found monoinfections of *P*. *vivax* and *P*. *falciparum*, as well as co-infections of both pathogens in diverse age groups (Table 1). In our study, 11 of 216 patients were presented with impaired consciousness (GCS <11 or BCS <3) during hospitalization, however they were excluded from the study because all 11 patients showed associated infections and comorbid illnesses such as pneumonia. Furthermore, all 11 patients were not identified by nPCR to have contracted malaria.

**Table 1:** Demographic characteristics of patients with *P*. *vivax* and *P*. *falciparum* infections, Orakzai Agency (FATA), Pakistan, 2011–2013^a^.

Characteristic		Frequency (%)		
		*P*. *vivax*	*P*. *falciparum*	Mixed
Sex	Male	112 (73)	12 (57)	1 (33)
	Female	42 (27)	9 (43)	2 (67)
Age	≤ 20	89 (58)	12 (57)	3 (100)
	> 20	65 (42)	9 (43)	0
Previously healthy individuals		147 (95)	21 (100)	3 (100)
Concurrent illness	Diabetes	7 (5)	0	0

^a^ Number of participants = 178 (diagnosed by nPCR and microscopy)

### Malaria diagnosis

**Fig 2:**
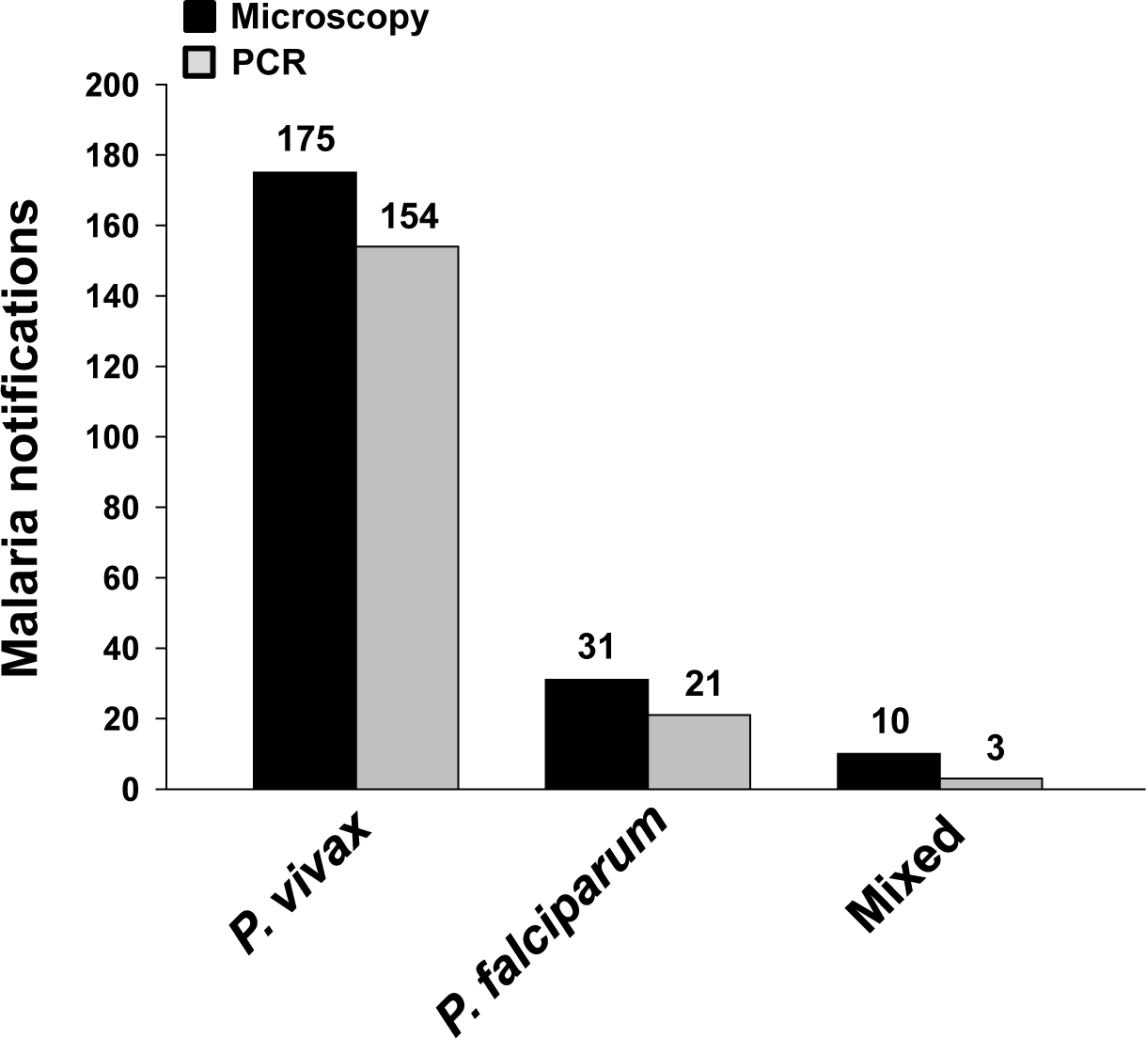
Microscopic and nPCR-based diagnosis of *Plasmodium* infections in clinical isolates showing different number of patients infected by *P*. *vivax*, *P*. *falciparum*, and mixed species (double infections with *P*. *falciparum* and *P*. *vivax*). *P*. *vivax* was found to be the most prevalent species.

The diagnosis was initially made by the microscopic examination and nPCR. The microscopic examination identified 175 patients (81%) to be infected by *P*. *vivax*, and 31 patients (14%) by *P*. *falciparum*, and 10 patients (5%) doubly infected by both *P*. *falciparum* and *P*. *vivax* (Fig 2). In contrast, the nPCR examination detected 154 (86%) *P*. *vivax* infections and 21 (12%) *P*. *falciparum* infections, respectively. This method detected 3 (2%) double infections. However, *P*. *ovale* and *P*. *malariae* infections were not identified in any of the investigated samples by both test methods. Noticeable discrepancies between microscopic (216 patients) and nPCR (178 patients) detections were observed (Fig 2). Previous comparative diagnosis studies demonstrated nPCR to produce sensitive and reliable diagnosis results better than other methods including microscopy [16, 17]. nPCR was acceptable to serve as the reference standard in malaria diagnosis. Therefore, we decided to rely on the nPCR diagnosis.

### Clinical characteristics

We observed greater prevalence of *Plasmodium* infection in males (70%). The further clinical and biochemical tests of patients with *P*. *vivax* and *P*. *falciparum* infections (Table 2) demonstrated the majority of the subjects (80%; n = 142 of 178) exhibiting severe malaria complications by World Health Organization criteria. As shown in Table 2, comparable and similar rates of various complications were observed in both *P*. *vivax* and *P*. *falciparum* patients. Among 121 febrile patients with severe *P*. *vivax* infection (Table 2), the frequency of complications was as follows: decompensated shock (n = 64; 53%; p = <0.001), hypoglycaemia (n = 12; 10%), anemia (n = 12; 10%), hyperpyrexia (n = 10; 8%), multiple convulsions (n = 10; 8%), hyperparasitaemia (n = 7; 6%), and jaundice (n = 6; 5%). On the other hand, the following frequency of complications was observed in febrile patients with severe *P*. *falciparum* infection (n = 21 of 178): decompensated shock (n = 9; 43%; p = <0.001), anemia (n = 4; 19%), jaundice (n = 3; 14%), multiple convulsions (n = 2; 9%), hypoglycaemia (n = 1; 5%), hyperpyrexia (n = 1; 5%), and hyperparasitaemia (n = 1; 5%). The frequency of complications among all patients who tested positive with malaria (n = 178) were as follows: decompensated shock (n = 73; 41%), anemia (n = 16; 9%), hypoglycaemia (n = 13; 7%), multiple convulsions (n = 12; 7%), hyperpyrexia (n = 11; 6%), jaundice (n = 9; 5%), hyperparasitaemia (n = 8; 5%), and mixed complications (n = 36; 20%) more than one criteria. The most common malarial complication caused by *P*. *vivax* and *P*. *falciparum* was decompensated shock (p = <0.001). Decompensated shock symptom had the highest odds ratio (OR) for being reported in patients affected by both malarial species (Table 2). Hypoglycemia, multiple convulsions and anemia had an OR in the similar range in case of *P*. *vivax* infected patients whereas a different pattern of OR was observed in *P*. *falciparum* infected patients (Table 2). Although all other statistical associations held, the strength of association varied.

**Table 2:** Comparison of complication rates in *P*. *vivax* versus *P*. *falciparum* infections, Orakzai Agency, Pakistan, 2011–2013^a^.

Complications	Case definition^b^	No. (%) *P*. *vivax* cases, n = 121	Odds ratio (CI)	p value	No. (%)*P*. *falciparum* cases, n = 21	Odds ratio (CI)	p value
Jaundice	Plasma or serum bilirubin >50 mM (>3.0 mg/dL) and parasite count >100, 000/μL	6 (5.0)	3.5 (0.7–17.9)	0.102	3 (14.3)	11.3 (1.7–72.4)	0.017
Hypoglycemia	Blood or plasma glucose concentration <2.2 mM (<40 mg/dL)	12 (9.9)	2.9 (1.0–8.5)	0.036	1 (4.8)	1.3 (0.1–11.9)	0.579
Decompensated Shock	Systolic blood pressure <70 mm Hg in children and <80 mm Hg in adults with evidence of impaired perfusion (cool peripheries or prolonged capillary refill)	64 (53.0)	21.0 (9.0–48.6)	<0.001	9 (42.9)	14.0 (4.4–44.3)	<0.001
Multiple convulsions	Generalized seizures (particularly in children), twitching of a digit, repetitive jerky eye movements with deviation, or increased salivation	10 (8.3)	2.4 (0.7–7.2)	0.092	2 (9.5)	2.8 (0.5–15.4)	0.232
Hyperparasitaemia	*P*. *falciparum* parasitaemia >10% of total red cells or lower parasitaemias in case of *P*. *vivax*	7 (5.6)	8.4 (1.0–69.3)	0.021	1 (4.8)	6.8 (0.4–113.9)	0.247
Anemia	A Hg concentration <5 g/dL in children or <7 g/dL in adults together with a parasite count >10,000/μL	12 (9.9)	2.4 (0.8–6.6)	0.065	4 (19.0)	5.1 (1.3–20.2)	0.028
Hyperpyrexia	Body temperature >38°C	10 (8.3)	1.2(0.5–3.3)	0.382	1 (4.8)	0.7 (0.08–5.9)	0.609

^a^ WHO, World Health Organization; CI, Confidence Interval; Hg, hemoglobin.

^b^ Case definition was based on the WHO criteria [14].

### Epidemiological characteristics

**Fig 3:**
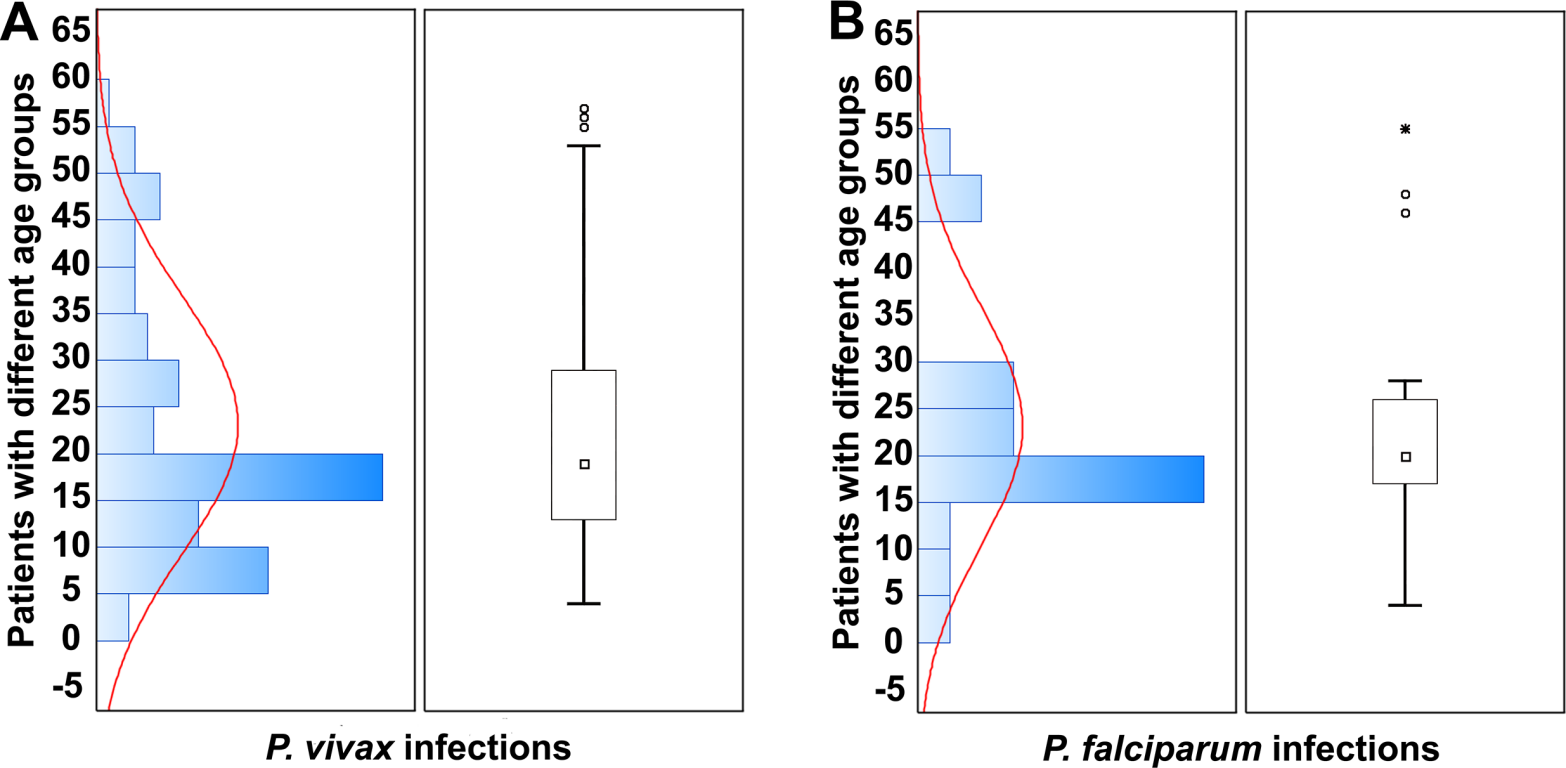
Graphical summary of attributable fractions of malarial infections caused by *P*. *vivax* (A) and *P*. *falciparum* (B) in different age groups of malarial patients. Data are presented as histograms (left panel with curve showing the pattern of *Plasmodium* infection incidence in different age groups), and box and whisker plots (right panel) showing median (□), lower quartile, upper quartile, outliers (○), and extreme score (*) of their respective sample distributions.

The mean parasite count for *P*. *vivax* patients was (20241.9; p = 0.744), which is significantly greater than that of *P*. *falciparum* patients (11848; p = 0.744). The mean illness duration was 5.2 ± 2.0 days for *P*. *falciparum* male patients and 4.4 ± 0.7 days for females. Similarly, the mean illness duration for *P*. *vivax* male patients was 5.5 ± 1.6 days and 6.1 ± 1.3 days for female patients. The incidence of both vivax and falciparum malaria gradually increased between the ages of 1–20 years with increasing age (Fig 3). The prevalence of malaria reached its peak among late teenagers (the age of 15–20, see Fig 3). It was also obvious that *P*. *vivax* infections were most prevalent in children of the age group between 5 and 15 years old as shown in Fig 3A, while *P*. *falciparum* infections in children populations were less prevalent as compared with *P*. *vivax* (Fig 3B). However, the most noteworthy characteristic was the drastic decrease in malaria incidence in post-puberty males and females (21–60 years). This age-dependency was observed in both parasite species infections (*P*. *falciparum* and *P*. *vivax*). High childhood malaria parasite exposure resulted in children (1–15 years) bearing the brunt of the disease burden (Fig 3). This group turned out to be the most vulnerable.

## Discussion

The malaria epidemic and endemic in Pakistan is a present and ongoing threat to public health which could have an impact in the nearby regions as well. In 2008, 2.6 million malaria cases were reported countrywide with a death rate of 50,000 per year [8, 18, 19]. National and provincial malaria control programs are dependent on a network of facilities providing diagnosis data. However, the regional situation due to the constant military conflicts and Talibanization of the Orakzai Agency in FATA collapsed the regional healthcare systems, and has made any epidemiological study practically impossible albeit essential for disease control and management.

For the first time, we carried out a reliable epidemiological and clinical study with febrile patients. In this study, 86.5% of the malaria cases were attributed to *P*. *vivax* infections, and 11.8%, to *P*. *falciparum* (p = 0.258). This result agreed with previous studies performed in nearby areas in Pakistan, as it was shown that the prevalence of *P*. *vivax* changes from 70% (30% *P*. *falciparum*) in mid Pakistan province to 90% (10% *P*. *falciparum*) in areas near or inside Afghanistan including FATA [8]. Moreover, we could not find any *P*. *ovale* and *P*. *malariae* infections in our febrile patient samples. This result also agreed with the fact that these two species had been negligible in this region [7, 20, 21]. To ensure that the results represented unbiased reality of the malaria endemic and epidemic in Orakzai Agency, all samples were collected from patients during the transmission season as the *P*. *vivax* transmission season peaks between April and September, while the *P*. *falciparum* peaks between August and December [7, 21]. *P*. *vivax* is the most prevalent in hilly areas, while *P*. *falciparum* has the lowest prevalence rate [22]; similarly, the Orakzai Agency is covered with hills and two rivers, making this area suitable for malarial parasites breeding.

We observed a relationship between patient sex and malarial infections. The predominance of malarial infection in male patients between the ages of 10 and 20 years was observed in our studies as well as in earlier investigations in Pakistan [22, 23]. It is believed that males have more outdoor exposure then females, and hence have increased vector exposure because they have to do laborious work in agricultural fields but are not as well-covered as adult females. In fact, in the Orakzai Agency women are totally covered with Burqa: an enveloping outer garment worn by women. As a result, they faced increased *Anopheles* bites. Therefore, there has been the disproportionate number of male malaria patients. On the other hand, during this study, we were intrigued to see that poor socioeconomic conditions together with the lack of public health infrastructure in Orakzai Agency might also be the cause of the disproportionateness. In Talibanized regions, women were often banned from traveling to a hospital without an accompanying male. In addition, in extreme poverty, a family may not afford medical attention to a sick woman. Indeed, comparable rate of malaria infections in both genders in other Pakistani provinces was observed in previous studies [22, 23].

In this study, we also observed children being excessively susceptible to malaria. Approximately half of the vivax malaria patients (n = 54) were children (≤20 years old) identified as having severe illness. This is consistent with other studies of hospitalized children in the South Asia region and in India [24]. This unequal susceptibility may possibly be attributed to the poor yet-to-develop immunity against the parasites [14]. As they get older and more exposed to the parasites, however, they can progressively develop adequate immunity to malaria.

In our study, clinical and laboratory analysis identified 80% of malaria patients (n = 142) as having severe illness (Table 2). Anemia has been the typical consequence of malaria, and we found 9% of malaria patients (n = 16 out of 178; p = 0.061) with severe anemia. This was more prevalent in *P*. *vivax* infected males (10%; n = 12 out of 121), while anemia induced by *P*. *falciparum* is considered more recurrent and more severe than anemia induced by *P*. *vivax*. Similar findings of prevalent *P*. *vivax*-induced anemia have also been reported previously [25–27]. In addition, 10% of patients infected by *P*. *vivax* (n = 12) were found to be with severe hypoglycemia. This result agreed with a previous report which had identified 9% of *P*. *vivax* malaria patients as having severe hypoglycemia [28, 29].

In our study, it is interesting to note that the manifestations of severe malaria instigated by *P*. *vivax* infection were more intricate than that of *P*. *falciparum* malaria. *P*. *vivax* malaria has reportedly caused tertian ague malaria that rarely led to a severe form [29]. However, many recent studies have shown an elevated risk of morbidity and mortality in *P*. *vivax* malaria [30–32]. Jaundice is one of the common manifestations of vivax malaria. In Northwestern India, jaundice has been reported in up to 57% of hospitalized patients infected by *P*. *vivax* [33]. In this study, jaundice was observed much less frequently, only in 5% *P*. *vivax*-infected patients (n = 6). We also found a similar prevalence of hyperpyrexia (8%; n = 10 out of 121) among *P*. *vivax*-infected patients to that of the previous study [34]. Cerebral malaria in patients with *P*. *vivax* infection was observed with multiple convulsions accounting for 8% of severely ill subjects, but not as frequently (14%) as seen in Bikaner, India [24].

Interestingly, the parasite count of *P*. *vivax* was very high in our studies. The lofty level of parasitic density of *P*. *vivax* as well as that of *P*. *falciparum* in some patients may presumably reflect the immune tolerance resulting from repeated exposures. Our study also reports the frequency of decompensated shock (41%; n = 73 out of a total of 178 patients; p = <0.001). This rate is the highest among similar studies reported previously. We found that decompensated shock was a common and prominent clinical feature of malaria caused both by *P*. *vivax* (53%; n = 64; p = <0.001) and *P*. *falciparum* (42.9%; n = 9; p = <0.001). Our result corresponds to the findings which also reported that *P*. *vivax* malaria could cause decompensated shock in infected individuals [23, 27].

### Conclusion

Nonetheless, our study has its own limitations including lack of meticulous refugee statistics. Our study failed to estimate the true number of imported malarial cases in Orakzai Agency. Due to the presence of the armed Taliban insurgents in the Upper Orakzai, we were unable to collect the disaggregated data of Orakzai Agency. However, this study is the first report on the epidemic situation and clinical analysis regarding this most neglected region. Furthermore, in our study, children were the most susceptible population to malaria infection whereas they were the least expected to use satisfactory prevention strategies in such a war-torn deprived region. Local health authorities should initiate malaria awareness programs in schools and malaria-related education should be further promoted at the local level reaching out to both children and parents. We conclude that this overlooked distribution of malaria should be considered by malaria control strategy makers in the world and by the Pakistani government.
